# Treatment Outcomes and Associated Factors in Tuberculosis Patients at Atwima Nwabiagya District, Ashanti Region, Ghana: A Ten-Year Retrospective Study

**DOI:** 10.1155/2021/9952806

**Published:** 2021-07-19

**Authors:** Sadick Ahmed Agyare, Francis Adjei Osei, Samuel Frimpong Odoom, Nicholas Karikari Mensah, Ernest Amanor, Charles Martyn-Dickens, Michael Owusu-Ansah, Aliyu Mohammed, Eugene Osei Yeboah

**Affiliations:** ^1^Ghana Health Service, Atwima Nwabiagya, Ghana; ^2^Kwame Nkrumah University of Science and Technology, Kumasi, Ghana; ^3^Komfo Anokye Teaching Hospital, Kumasi, Ghana; ^4^Ghana Health Service, Zuarungu, Ghana

## Abstract

**Introduction:**

Tuberculosis poses a great threat to public health around the globe and affects persons mostly in their productive age, notwithstanding; everyone is susceptible to tuberculosis (TB) infection. To assess the effectiveness and performance of the tuberculosis control program activities, the percentage of cases with treatment success outcome is key. To control tuberculosis, interrupting transmission through effective treatment cannot be overemphasized. The study was conducted to determine factors associated with TB treatment outcome, in the Atwima Nwabiagya District from 2007–2017.

**Method:**

A Retrospective review of routine/standard TB registers was carried out in five directly observed therapy short-course (DOTS) centres at the Atwima Nwabiagya District from January 2007 to December 2017. Demographic characteristics, clinical characteristics, and treatment outcomes were assessed. Bivariate and multivariate logistic regression was conducted to determine the predictors of successful treatment outcome.

**Results:**

Of the 891 TB client's data that was assessed in the district, the treatment success rate was 68.46%. Patients, aged ≤ 20 years (adjusted odds ratio (aOR) = 4.74, 95%CI = 1.75 − 12.83) and 51-60 years (aOR = 1.94, 95%CI = 1.12 − 3.39), having a pretreatment weight of 35-45 kg (aOR = 2.54, 95%CI = 1.32 − 4.87), 46-55 kg (aOR = 2.75, 95%CI = 1.44 − 5.27) and 56-65 kg (aOR = 3.04, 95%CI = 1.50 − 6.14) were associated with treatment success. However, retreatment patients (aOR = 0.31, 95%CI = 0.11 − 0.84) resulted in unsuccessful treatment outcome.

**Conclusion:**

Successful treatment outcome among TB patients was about 20.00% and 30.00% lower compared to the national average treatment success rate and WHO target, respectively. Active monitoring, motivation, and counselling of retreatment patients and patients with advanced age are key to treatment success.

## 1. Background

Tuberculosis (TB) remains one of the oldest debilitating infectious diseases globally but disproportionately affect the world's poor. The clinical presentation of TB is associated with the pathogenesis of an Acid-Fast Bacilli bacterium called *Mycobacterium tuberculosis* which is transmitted through aerosols or droplets when an individual with an active infection coughs, sneezes, sings, or talks [1, 2]. Persons infected by *Mycobacterium tuberculosis* may not develop symptoms of TB (latent TB); nonetheless, infected individuals have a 5-15% lifetime risk of becoming sick within 2-5 years of getting the infection [3–5]. However, individuals who are immunocompromised such as people with HIV, diabetes, malnutrition, or heavy smokers, and older adults and children have a higher risk of developing symptoms of TB [4]. This may include a cough that produces phlegm, fever, chills, fatigue, loss of appetite, and weight loss. It is reported that a single TB patient with active infection can infect an average of 10-15 people annually if the patient is not treated [6].

Approximately 10 million cases of TB and 1.5 million deaths are recorded every year globally [7]. Out of the 30 countries with the highest burden of TB globally, 24 are in the WHO African region [8, 9]. Nearly 2.5 million people who contracted TB, 665,000 of them died from the disease in 2017 in sub-Saharan Africa [9]. Analysis of global TB data shows that the annual TB incidence has been on a downturn by an average of 1.5%, and the management of TB has significantly improved since 2000 [8]. Interestingly, about 49 million lives have been rescued as a result of early and improved TB diagnosis and appropriate treatments between 2000 and 2015 [5]. Despite the mammoth progress of global efforts, TB remains one of the leading causes of morbidity and mortality worldwide and remains a substantial public health burden in sub-Saharan Africa [10]. It is reported that the prevention, care, and control of TB has been challenging due to socioeconomic reasons [10]. Therefore, it is imperative to scale-up management efforts to meet the End TB strategy, which aims at reducing TB mortality rate by 90% by the year 2030 [11].

Successful treatment outcomes of TB vary from country to country but 83% of treatment outcomes have been attained worldwide [12]. Nonetheless, several studies have reported age, biological sex, comorbidity or underlining health conditions, pretreatment weight, and financial status or family support as factors that influence treatment outcomes of TB in sub-Saharan Africa [13, 14]. Comorbidity with HIV was found to be strongly associated with TB treatment outcomes, and apparently, treatment outcomes were best for individuals with a negative HIV status compared with those positive for HIV [12, 13, 15]. Moreover, most studies report that being male, older adults, and large family size are associated with unsuccessful treatment outcomes in sub-Saharan Africa [15, 16]. According to WHO, about 6 million adult men contracted TB and around 840,000 died from it compared with roughly 3.2 million and nearly half a million adult women who fell ill and died from TB, respectively, in 2017 [8]. However, the factors influencing treatment outcome may vary from one setting or region to another. For instance, a study reported a successful treatment outcome rate of 82.5% in the Volta region, 90.2% in the Central region, and 90.7% in the Greater Accra region of Ghana [17, 18].

In Ghana, over 46,000 new cases of active TB infection are recorded yearly, and TB mortality rate is reported at 7.5 per 1,000 infected people, indicating that TB is a burden in the country [5, 19]. Successful treatment outcomes are very crucial to mitigate the burden of TB of which several studies have reported successful outcome rates nearly similar to the national successful treatment outcome rate (87.7%) [17, 20, 21]. Furthermore, factors such as HIV comorbidity, biological sex, disease category, patient category, financial status, the distance of patient from the treatment centre, among others were found to be associated with treatment outcomes [20, 21]. Successful treatment outcomes were associated with females, HIV-negative status, high financial index, and the proximity of patients to treatment centres. However, pretreatment weight varied [17, 18, 20, 22].

Evaluating the treatment outcome of TB cases and its determinants is important to inform health authorities and policymakers on the management of the disease. However, studies exploring TB treatment outcomes and the associated factors are limited in Ghana and lacking in many districts in the Ashanti Region of Ghana. Given the paucity of evaluation data on TB treatment outcome in the Ashanti Region, it is essential to conduct this study to contribute to the body of knowledge and provide health managers and policy makers with evidence necessary to improve the current situation.

Therefore, this study is aimed at assessing treatment outcomes of TB and the factors that influence the observed treatment outcomes in TB patients at the Atwima Nwabiagya District, Ashanti Region, Ghana.

## 2. Materials and Methods

### 2.1. Study Area

The study was conducted in the Atwima Nwabiagya district in the Ashanti Region of Ghana. The district is perched approximately on latitude 6°32′N and 6°75′N and longitude 1°45′W and 2°00′W. It covers an estimated area of 294.84 square km with a population of 176,171 and is situated in the western part of the Ashanti Region. The five DOTS centres in the Districts that were selected are Nkawie-Toase Hospital, Abuakwa Health Centre, Akropong Health Centre, Asuofua Health Centre, and Barekese Health Centre.

### 2.2. Study Design

A retrospective review of standard TB registers was conducted from January 2007 to December 2017 to determine successful TB treatment outcome and associated factors among TB patients.

### 2.3. Study Population

All patients diagnosed with and treated with TB and had treatment outcomes in the TB registers were included in the study whereas patients who were referred or entries with incomplete information on treatment outcomes were excluded.

### 2.4. Sample Size Determination and Sampling Technique

The sample size was determined by using a single population prevalence formula.

(1) $$\begin{array}{c} n=\frac{{\left(Z_{\alpha }+Z_{\beta }\right)}^{2}p\left(1-p\right)}{d^{2}}, \end{array}$$

where *n* is the minimum required sample size. *Z*_*α*_ is the desired level of statistical significance: 95% confidence level which translate to 1.96 to a standard normal deviate of 1.96. *Z*_*β*_ is the desired power of the test: 80% which translates to 0.84 standard normal deviate. *p* is the prevalence of successful treatment outcome of TB patients from previous study: 82.5% [22]. *d* is the margin of error: 4%.

Substituting the above values into the formula;

(2) $$\begin{array}{c} n=\frac{{\left(1.96+0.84\right)}^{2}0.825\left(1-0.825\right)}{0.04^{2}}, \end{array}$$

(3) $$\begin{array}{c} n=707.43, \end{array}$$

(4) $$\begin{array}{c} n\approx 707. \end{array}$$

After accounting for a 20% nonresponse rate of missing data, the minimum sample size was 848.

Study participants who met the inclusion criteria were selected from the standard TB registers through convenient sampling technique at the 5 DOTS centres.

### 2.5. Data Collection, Data Quality, and Data Analysis

Data were collected from the standard TB registers (the TB registers are used to capture information of all TB patients been treated at all DOTS centres in the districts) using a pilot-tested data abstraction sheet. The abstraction sheet was designed electronically using Open Data Kit (ODK). The sheet was pilot tested at Komfo Anokye Teaching Hospital's (KATH), TB Clinic, which provides similar services to TB patients as the study site. The data were collected by trained research assistants. The data were reviewed by a data manager for completeness and discrepancies. Information such as demographic characteristics, clinical characteristics, and treatment outcomes were collected from the TB registers.

The data were exported from the ODK to Microsoft Excel, cleaned, and exported to Stata/SE 14.0 statistical software (Stata Corp 4905 Lakeway Drive College Station, Texas 77845, USA) for analysis. Descriptive statistical analysis such as mean, standard deviation, and frequencies (percentages) was used to describe the study population. Bivariate and multivariate logistic regression analysis was done with 95% confidence interval (CI) to calculate the crude odds ratio (OR) and adjusted odds ratio (aOR) to measure the strength of the association between the outcome (treatment outcome) and predictor variables (age, gender, pretreatment weight, HIV status, diseases classification, type of patient, availability of treatment supporter, and adverse drug reaction).

### 2.6. Operational Definitions and Treatment Regimen

Table 1 displays the operational terms used in this study. The operation was based on the standard definitions adopted from the World Health Organization guideline [7].

The recommended DOTS treatment regimens are guidelines for each category of TB in Ghana by the National TB Control Program and the National AIDS Control Program [23] (Table 2).

### 2.7. Ethical Considerations

Ethical approval for the conduct of the study was obtained from the ethical review board of the Ghana Health Service Research and Development Division (Ref. No. GHS-ERC: 76/12/18). Permission was also sought from the Ashanti Regional Health Directorate, Atwima Nwabiagya District Health Directorate, and the five DOTS centres. The information obtained from the TB registers was used for only research purposes and is kept confidential in accordance with the declaration of Helsinki (1964). No identification information such as names was extracted or captured from the records.

## 3. Results

### 3.1. Background Characteristics of the TB Patients

**Figure 1:**
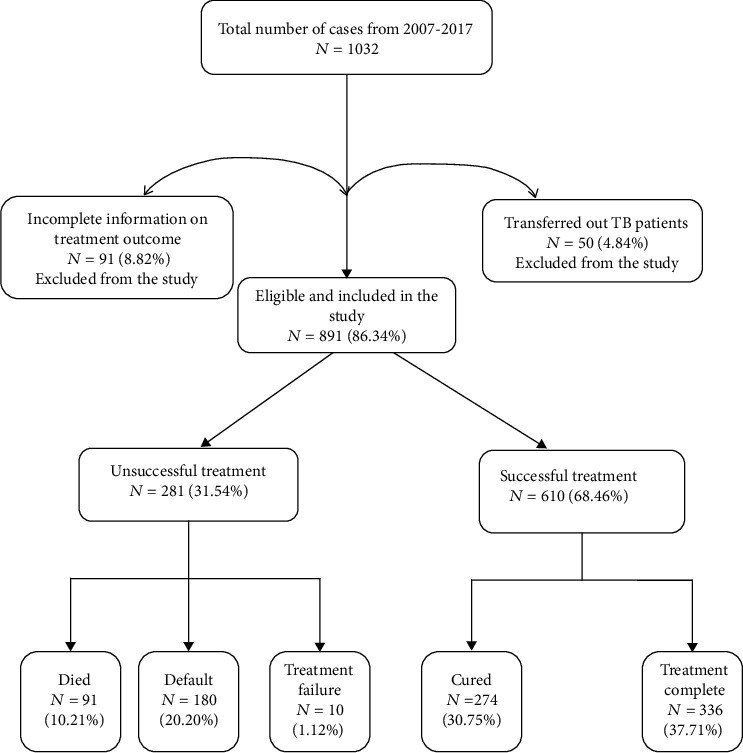
Flowchart of TB treatment outcome in Atwima Nwabiagya District, Ashanti Region, Ghana from 2007-2017.

A total of 1,032 TB patients were registered at the 5 DOT centres at the Atwima Nwabiagya District during the period (2007-2017) of review. Of these, 891 (86.34%) patients had complete data and were therefore included in the study (Figure 1). More than half 599 (67.23%) of the patients were females. The age distribution of the patients ranged from 12–95 years, with a mean and standard deviation of 46.63 and 16.24 years, respectively. Nearly one-quarter 217 (24.35%) were within the ages of 31-40 years. Before the initiation of the regimen, 260 (29.18%) of the patients weighed 36-45 kg with a mean and standard deviation of weight as 49.64 kg and 9.42 kg, respectively. HIV/TB coinfection was observed in 82 (9.20%) of the patients. More than half, 535 (60.04%), of the patients were classified as extrapulmonary TB and more than three quarters of 793 (89.00%) were newly diagnosed with TB. Few 4 (0.45%) of the patients had adverse reactions to the TB regimen (Table 3).

### 3.2. Treatment Outcomes of TB Patients

The proportion of death among the TB patients was 10.21%. A total of 366 (37.71%) and 274 (30.75%) of the patients completed their treatment and were cured, respectively, giving a successful treatment rate of 68.46% (*n* = 610). Treatment failure was observed among 10 cases (1.12%) and a default rate of 20.2% (*n* = 180). Unsuccessful treatment was observed in 281 (31.54%) of them (Table 4).

### 3.3. Factors Associated with Treatment Outcome

Unadjusted logistics regression analysis revealed that the odds of achieving a successful treatment outcome was 5.57 (95%CI = 2.08 − 14.90, *p* ≤ 0.001) and 2.06 (95%CI = 1.20 − 3.53, *p* = 0.009) higher among patients aged at less or equal to 20 years and 51-60 years compared to their age counterparts. Patients with weights between 36-45, 46-55, and 56-65 kg were 2.54, 2.50, and 2.79 times more likely to develop treatment success compared to patients with weight greater than 65 kg, respectively ((OR = 2.54, CI = 1.36 − 4.74, *p* = 0.004), (OR = 2.50, CI = 1.36 − 4.59, *p* = 0.003), (OR = 2.79, CI = 1.44 − 5.40, *p* = 0.002)) (Table 5)

Adjusted logistic regression revealed that patients aged less or equal to 20 years and 51-60 years were 4.74 and 1.94 times more likely to develop treatment success as compared to other age groups ((aOR = 4.74, CI = 1.75 − 12.83, *p* ≤ 0.002), (aOR = 1.94, CI = 1.12 − 3.39, *p* = 0.019)). Patients weight between 36-45, 46-55, and 56-65 kg were 2.43, 2.49, and 2.75 times more likely to develop treatment success as compared to patients with weight greater than 65 kg ((aOR = 2.54, 1.32-4.87, *p* = 0.005), (aOR = 2.75, CI = 1.14 − 5.27, *p* = 0.002), (aOR = 3.04, CI = 1.50 − 6.14, *p* = 0.002)). Retreatment patients were 69.0% less likely to develop treatment success (OR = 0.31, CI = 0.11 − 0.84, *p* = 0.021) (Table 5).

## 4. Discussion

Improved diagnosis and successful treatment of TB have been instrumental in mitigating and averting millions of TB deaths annually. However, TB treatment programs in sub-Saharan Africa are faced with several challenges that make these programs not as highly effective as expected, and thus, these factors result in unsuccessful treatment outcomes. We evaluated the TB treatment outcomes and the predictors of successful and unsuccessful treatment outcomes which is very key to the performance of a National TB control program. The findings of this study show that most TB clients were either cured or completed treatment; hence, the rate of successful treatment outcome in the study areas was 68.46%. Though majority of the patients responded to the treatment, the rate of treatment success was below the national treatment success rate of 87.7% [16, 20] and rates recorded in other parts of the country [17, 18, 22] as well as rates in other countries like Ethiopia (Southwest 85.9%, Eastern 92.5%) [12, 24], Kenya (82.4%) [25], and Iran (91.7%) [26]. However, our finding is similar to the rate of treatment success reported by a study in South Africa (70%) [14].

The difference in treatment success observed in this study compared to other previous studies could be due to variations of the study duration, which was usually 5 years for most studies, and sample size, which was relatively low given the duration of this study [12, 24, 25]. The unsatisfactory treatment outcome can also be explained by the high number of defaulters, few participants who died, and the very few who failed treatment which was higher in the study compared to other previous studies [12, 24, 25]. This probably implies patient follow-up or treatment supervision has been poor in the district or patient delay in seeking TB care as a result of stigma or inadequate community involvement in TB care. Furthermore, patients who failed treatment might have developed resistance to anti-TB medication. Therefore, it is important to ensure rigorous treatment supervision and to engage all stakeholders in patient care through the community TB care, an intervention that recognizes social capital and connectedness as a tool to effective TB treatment and control.

Globally, TB morbidity and mortality are significantly higher among men than women, and it is reported that the ratio of male to female TB mortality and/or morbidity is about 2 : 1 in low-and middle-income countries [27, 28]. Though the cause of this observation is not clearly understood, it is believed that epidemiological factors could be the impetus driving this trend [29]. Studies have revealed that men are less likely to have a timely TB diagnosis due to the fear of stigmatization, and, within the context of HIV, men are likely to default their treatment and experience worse outcomes [28, 30]. On the other hand, women are more likely to seek timely diagnosis but due to socioeconomic constraints and stigmatization, many resort to orthodox or home treatment [26, 31]. Contrary to previous studies, our findings revealed that most of the reported TB cases were females compared to males, and the rate of treatment success was nearly the same for both genders. Nonetheless, there was no significant association between gender and treatment outcomes. This may suggest that women access diagnostic and screening services more than men or men are at a disadvantage in seeking and accessing TB care.

The majority of the respondents are between the ages range 31-40 years and 41-50 years, and this was consistent with a few studies in Ghana [17, 22, 32]. This suggests that individuals of the productive age group are mostly infected by TB in the district. However, the odds of successful treatment outcomes were better for respondents at age less or equal to 20 years (5.57) and 51-60 years (2.06). Based on this evidence, the economy could be burdened because the majority of the TB patients fall within the age group who might not be able to work and support the nonworking force due to the disease. Given that TB mostly affects individuals of the productive age group but less likely to have treatment success demands that community TB care must be pursued vigorously in the district. According to our findings, pretreatment weight between 36-45 kg, 46-55 kg, and 56-65 kg were significantly associated with successful treatment outcome compared to those less than 35 kg and greater than 65 kg. Furthermore, pretreatment weight between 36-45 kg, 46-55 kg, and 56-65 kg were more likely to record successful treatment outcomes. Although the effect of pretreatment weight differences on treatment outcome varies between settings, our finding is similar to the outcomes of a study conducted in the eastern part of Ethiopia [12]. TB is a wasting disease, and it has been shown that bodyweight variations can predict TB treatment outcome [33]. Thus, TB patients losing weight during TB treatment particularly within the first two months are likely to fail treatment [33, 34]. This is because as they lose weight, the dosage of drugs has to be reduced to avert side effects, and as result, this may reduce the efficacy of the drug to kill *M. tuberculosis*.

Unlike several studies that reported a strong association between HIV comorbidity and treatment outcome [21, 27, 35], this study recorded an insignificant association between treatment outcome and HIV comorbidity. Likewise, the majority of the patients reviewed were negative for HIV which contradicts the status quo of high preponderance of HIV among TB patients [36, 37]. These differences might be a result of the poor reading of HIV test results or insensitive HIV test kits; however, this could not be validated since the study was based on the retrospective review of patients' documents. In this study, majority of the TB patients were extra-PTB and smear-negative PTB compared to smear-positive and MDR-TB which was consistent with several studies in other parts of Ghana and other countries. However, extra-PTB and smear-negative PTB were not associated with treatment outcome which contravenes with several other studies. The high proportion of extra-PTB and smear-negative PTB cases recorded might be due to overdiagnosis or misdiagnosis or misclassification bias since these disease categories are diagnosed only based on the clinical condition of the patient. Moreover, it is assumed some suspected TB cases might have been diagnosed first in one health facility and transferred to another health facility; hence, the clinicians might overlook the sputum test and might rush to diagnose the patient as extra-PTB or smear-negative PTB. This in effect could affect treatment outcomes; therefore, we suggest sputum test plus clinical condition as in the case of smear-positive PTB for all disease categories during monitoring of treatment outcome.

Most of the TB cases were new cases, and most of the patient categories were likely to be successful with treatment. Nevertheless, patients on retreatment were less likely to be successful with treatment. Similar findings from other studies in Ghana [38, 39], South Africa [40, 41], Ethiopia [42, 43], and Kenya [29] also reported that retreatment was significantly associated with unsuccessful treatment outcome. The reasons for these observations might be that as the patients were continually exposed to the anti-TB medication, suboptimal therapy and drug resistance might have occurred.

## 5. Conclusion

This study found an overall treatment success rate of 68.46%, which was lower compared to the national average treatment success rate of 87.0% and 90.0% target for WHO. It was deduced that the contributing factors to successful treatment outcome were age, pretreatment weight, and patient category including those who relapse, transfer in, failure of treatment, and retreatment. Based on the findings of this study, we recommend active monitoring, motivation, and counselling of TB patients during treatment to avoid defaulting of treatment procedures especially in those advanced in age.

### 5.1. Limitation of This Study

The major limitation to our study is the use of retrospective secondary data. Variables such as comorbidities (diabetes, hypertension, etc.), distance to health facility, income level, treatment- and disease-related factors (drug resistance and adherence), behavioural factors (knowledge and attitude about TB), and smoking and alcohol history were not routinely captured in the TB registers.

Also, incomplete data and transferred out patients might have had an impact on treatment outcome or the results of the study.

## Figures and Tables

**Table 1:** Operational definitions used in this study.

Term	Definition
Cured	A pulmonary positive TB patient who has completed a full course of treatment and has tested negative to smear or culture-negative in the last month of treatment and at least one previous occasion.
Treatment completed	A TB patient who completed treatment without failure of treatment and with no evidence to show that sputum smear or culture results in the last month of treatment and on at least one previous occasion were negative, either because tests were not done or because results are unavailable.
Treatment failure	A TB patient who remains sputum smear or culture positive at month five or later during treatment regardless of adherence to the prescribed treatment protocols
Died	A TB patient who dies for any reason before starting or during treatment.
Default	A patient who interrupted treatment consecutively for two weeks or more after initiation of treatment
Transferred in	A patient who has been transferred from another TB register to continue treatment transferred to another reporting and recording unit for continuation of treatment, for whom treatment outcome is unknown.
HIV infection	Being infected with the human immune deficiency virus (HIV) that is confirmed by first and second line serologic tests
HIV/TB coinfection	The presence of both HIV and TB infection in a patient.
Successful treatment	A combination of cure and treatment completed.
Unsuccessful treatment	A combination of death, default, and treatment failure
New patient	A patient who has never had treatment for TB or who has taken anti-TB drugs for less than 1 month
Relapse	A patient previously treated for TB declared cured or treatment completed and who is diagnosed bacteriological (+) TB (smear or culture).
Failure	A patient who is restarted on a TB treatment after having failed previous treatment
Treatment supporter	A person who assists the patient in following treatment schedule throughout treatment. It can be a relative or a health professional

**Table 2:** TB treatment regimen.

Patient category	Definition	Initial phase treatment^∗^	Continuous phase treatment
		Daily (28 doses/month)	Daily (28 doses/month)
All new clients	New smear-positive	2 (HRZE)^¥^ = 56 doses of HRZE	4 (HR) = 112 doses of HR
	New smear-negative pulmonary TB		
	Concomitant HIV disease		
	Extrapulmonary TB		

Previously treated sputum smear-positive pulmonary TB	Relapse	2 (HRZE)S + 1(HRZE) = 84 doses of HRZE + 56 doses of S	5 (HRE) = 140 doses of HRE
	Treatment after interruption		
	Treatment failure		

Childrenª	Children below 12 years	2 (HRZ) = 56 doses of HRZ	4 (HR) = 112 doses of HR

^∗^Direct observation of treatment intake is required and always in regimen including rifampicin. ^¥^Streptomycin may be used instead of ethambutol. In meningitis, ethambutol should be replaced by streptomycin. ªIn children with meningitis, add streptomycin in the initial phase. Codes for Tb drugs: H: isoniazid; R: rifampicin; Z: pyrazinamide; S: streptomycin; E: ethambutol. Fixed drug combinations (FDC) codes: (HR): isoniazid + rifampicin; (HRZ): isoniazid + rifampicin + pyrazinamide; (HRZE): isoniazid + rifampicin + pyrazinamide + ethambutol.

**Table 3:** Background characteristics of TB patients.

Variable	Frequency (*n* = 891)	Percentage (%)
Gender		
Female	599	67.23
Male	292	32.77
Age (years)		
≤20	48	5.39
21-30	150	16.84
31-40	217	24.35
41-50	214	24.02
51-60	117	13.13
>60	145	16.27
Mean (SD)	43.63 (±16.24)	
Pretreatment weight (kg)		
≤35	48	5.39
36-45	260	29.18
46-55	366	41.08
56-65	164	18.41
>66	53	5.95
Mean (SD)	49.64 (9.42)	
HIV status		
Negative	809	90.80
Positive	82	9.20
Disease classification		
Smear positive PTB	85	9.54
Smear negative PTB	270	30.30
Extra-PTB	535	60.04
MDR-TB	1	0.11
Patient category		
New	793	89.00
Relapse	53	5.95
Transfer in	8	0.90
Failure	18	2.02
Retreatment	19	2.13
Treatment support		
No	89	9.99
Yes	802	90.01
Adverse drug reaction		
No	887	99.55
Yes	4	0.45

**Table 4:** Distribution of treatment outcomes of the TB patients.

Variable	Frequency (*n* = 891)	Percentage (%)
Successful	610	68.46
(i) Cured	274	30.75
(ii) Treatment complete	336	37.71

Unsuccessful	281	31.54
(i) Died	91	10.21
(ii) Defaulted	180	20.20
(iii) Treatment failure	10	1.12

**Table 5:** Bivariate and multivariate analysis of factors associated with successful treatment.

Variable	OR (95% CI)	*p* value	aOR (95% CI)	*p* value
Age (years)				
(i) ≤20	5.57 (2.08-14.90)	<0.001	4.74 (1.75-12.83)	0.002
(ii) 21-30	1.19 (0.74-1.90)	0.480	1.22 (0.74-2.00)	0.428
(iii) 31-40	1.48 (0.95-2.30)	0.081	1.47 (0.92-2.35)	0.103
(iv) 41-50	1.28 (0.82-1.99)	0.273	1.31 (0.83-2.07)	0.251
(v) 51-60	2.06 (1.20-3.53)	0.009	1.94 (1.12-3.39)	0.019
(vi) >60	Ref		Ref	

Gender				
(i) Male	Ref		Ref	
(ii) Female	0.86 (0.64-1.17)	0.350	0.76 (0.55-1.06)	0.111

Pretreatment weight (kg)				
(i) ≤35	Ref		Ref	
(ii) 36-45	2.54 (1.36-4.74)	0.004	2.54 (1.32-4.87)	0.005
(iii) 46-55	2.50 (1.36-4.59)	0.003	2.75 (1.44-5.27)	0.002
(iv) 56-65	2.79 (1.44-5.40)	0.002	3.04 (1.50-6.14)	0.002
(v) >65	1.66 (0.75-3.65)	0.210	1.78 (0.78-4.04)	0.170

HIV status				
(i) Negative	Ref		Ref	
(ii) Positive	0.69 (0.44-1.11)	0.127	0.68 (0.41-1.11)	0.119

Disease classification				
(i) Smear positive PTB	Ref		Ref	
(ii) Smear negative PTB	0.86 (0.51-1.47)	0.583	0.83 (0.48-1.45)	0.518
(iii) Extra-PTB	0.91 (0.55-1.50)	0.713	0.89 (0.53-1.50)	0.668
(iv) MDR-TB	—	—	—	—

Patient category				
(i) New	Ref		Ref	
(ii) Relapse	0.63 (0.35-1.11)	0.110	0.60 (0.34-1.08)	0.090
(iii) Transfer in	3.13 (0.38-25.57)	0.287	3.00 (0.34-26.54)	0.322
(iv) Failure	2.24 (0.64-7.79)	0.207	1.90 (0.52-6.90)	0.329
(v) Retreatment	0.40 (0.16-1.00)	0.051	0.31 (0.11-0.84)	0.021^∗^

Treatment support				
(i) No	Ref		Ref	
(ii) Yes	1.05 (0.66-1.68)	0.823	0.92 (0.56-1.51)	0.740

## Data Availability

Data supporting these findings of this study are available from the corresponding author.
